# PRACT: a pragmatic randomized adaptive clinical trial protocol to investigate a culturally adapted brief negotiational intervention for alcohol use in the emergency department in Tanzania

**DOI:** 10.1186/s13063-022-06060-y

**Published:** 2022-02-05

**Authors:** Catherine A. Staton, Siddhesh Zadey, Paige O’Leary, Ashley Phillips, Linda Minja, Monica H. Swahn, Jon Mark Hirshon, Judith Boshe, Francis Sakita, Joao Ricardo Nickenig Vissoci, Blandina T. Mmbaga

**Affiliations:** 1 Duke Global Health Institute, Duke University, 310 Trent Dr, Durham, North Carolina USA; 2 Division of Emergency Medicine, Department of Surgery, School of Medicine, 2301 Erwin Road, Durham, North Carolina USA; 3 Kilimanjaro Clinical Research Institute, Moshi, Tanzania; 4 Wellstar College of Health and Human Services, Kennesaw State University, Kennesaw, Parliament Garden Way Georgia 520 USA; 5 University of Maryland Baltimore Campus, 620 W Lexington St, Baltimore, Maryland United States of America; 6 Kilimanjaro Christian Medical Center, Moshi, Tanzania; 7 Kilimanjaro Christian Medical University College, Moshi, Tanzania

**Keywords:** Alcohol drinking, Binge drinking, Harm reduction, Pragmatic clinical trial, Randomized controlled trial, Wounds and injuries, Low-income populations

## Abstract

**Background:**

Alcohol use in resource-limited settings results in significant morbidity and mortality. These settings lack the necessary specialty-trained personnel and infrastructure. Therefore, implementing evidence-based interventions from high-income settings, like a brief negotiational intervention (BNI) for alcohol, will require rapid production of evidence of effectiveness to guide implementation priorities. Thus, this study describes a clinical trial protocol to rapidly optimize and evaluate the impact of a culturally adapted BNI to reduce alcohol-related harms and alcohol consumption among injury patients.

**Methods:**

Our pragmatic, adaptive, randomized controlled trial (PRACT) is designed to determine the most effective intervention approach to reduce hazardous alcohol use among adult (≥18 years old) in acute (< 24 h) injury patients. Our culturally adapted, nurse-delivered, intervention (PPKAY) has been augmented with evidence-based, culturally appropriate standards and will be evaluated as follows. Stage 1 of the trial will determine if PPKAY, either with a standard short-message-service (SMS) booster or with a personalized SMS booster is more effective than usual care (UC). While optimizing statistical efficiency, Stage 2 drops the UC arm to compare the PPKAY with a standard SMS booster to PPKAY with a personalized SMS booster. Finally, in Stage 3, the more effective arm in Stage 2 is compared to PPKAY without an SMS booster. The study population is acute injury patients who present to Kilimanjaro Christian Medical Centre, Tanzania, who (1) test alcohol positive by breathalyzer upon arrival; (2) have an Alcohol Use Disorder Identification Test of 8 or above; and/or (3) have reported drinking alcohol prior to their injury. Outcome measures will be evaluated for all arms at 3, 6, 9, 12, and 24 months. The primary outcome for the study is the reduction of the number of binge drinking days in the 4 weeks prior to follow-up. Secondary outcomes include alcohol-related consequences, measured by the Drinker Inventory of Consequences.

**Discussion:**

The findings from this study will be critically important to identify alcohol harm reduction strategies where alcohol research and interventions are scarce. Our innovative and adaptive trial design can transform behavior change research and identify the most effective nurse-driven intervention to be targeted for integration into standard care.

**Trial registration:**

ClinicalTrials.govNCT04535011. Registered on September 1, 2020.

**Supplementary Information:**

The online version contains supplementary material available at 10.1186/s13063-022-06060-y.

## Introduction

### Background and rationale

Alcohol causes over 3 million deaths annually [1]. In sub-Saharan Africa, alcohol is the leading avoidable risk factor, accounting for a substantial portion of the burden of death and disability [2, 3]. High rates of alcohol consumption have been associated with globalization in combination with rapid urbanization, economic development, increased availability of alcohol, corporate targeting, and weak policy infrastructure [4–8]. Drinking patterns in the sub-Saharan African region are the second-worst globally, with high rates of binge drinking and alcohol dependence [9]. Alcohol use, both binge drinking and chronic use, has been associated with many high-risk behaviors, including crime, aggressive driving, interpersonal violence, unintentional injuries, and self-inflicted injury [9]. Despite the high burden of alcohol-related harm across sub-Saharan Africa, research and interventions remain relatively scarce.

In high-income countries, brief negotiational interventions (BNI) for alcohol administered in an emergency department (ED) setting have shown to be both successful and cost-effective in reducing alcohol use more than 1-year post-intervention [10, 11]. However, in sub-Saharan Africa, individual-level interventions remain scarce [12]. Recent systematic reviews assessing randomized controlled trials of BNI for alcohol failed to include any studies from developing or transitioning countries [4, 13]. While there have been a few trials evaluating BNIs in Africa, research has focused on particularly high-risk populations, like female sex workers or low-income youth, and these trials have found variable success at reducing alcohol use [14]. The best BNI implementation strategy has also been controversial in the literature; integrating an intervention into clinical care can be challenging in the fast-paced, limited resource ED setting and the best practices of post visit “reminders” or “boosters” are not yet fully delineated. Some have found “boosters” or intermittent post-intervention reminders to be helpful, but others have found limited improvement in outcomes [15–17]. Similarly, integrating mobile health technology into a BNI appears promising, but in a global context, significant resource and cultural challenges limit potential implementation and effectiveness [18–21]. Finally, personalized boosters recounting the specific reasons for a patient's behavior change determined during the initial BNI have shown increased effectiveness at little to no increased cost [22, 23].

Conducting successive randomized clinical trials to determine the most effective intervention package would be cost-prohibitive and delay the implementation of a much-needed treatment option. Instead, using a pragmatic, randomized, adaptive clinical trial (PRACT), we posit that this reproducible trial method could be both efficient and cost-effective in defining and demonstrating the effectiveness of a BNI in a low-resource setting [24]. As such, we propose the PRACT to determine the effectiveness of this alcohol harm reduction intervention in three adaptive stages. This Phase II-III adaptive clinical trial will evaluate the effectiveness of a BNI to reduce alcohol use and alcohol-related harms when administered by nurses in an ED in a low-income setting. The goal of this article is to share the research protocol with the hope that it will facilitate the implementation of other adaptive trials seeking to reduce the burden of alcohol use and related harm in other low-resource settings.

### Trial Design

**Fig. 1:**
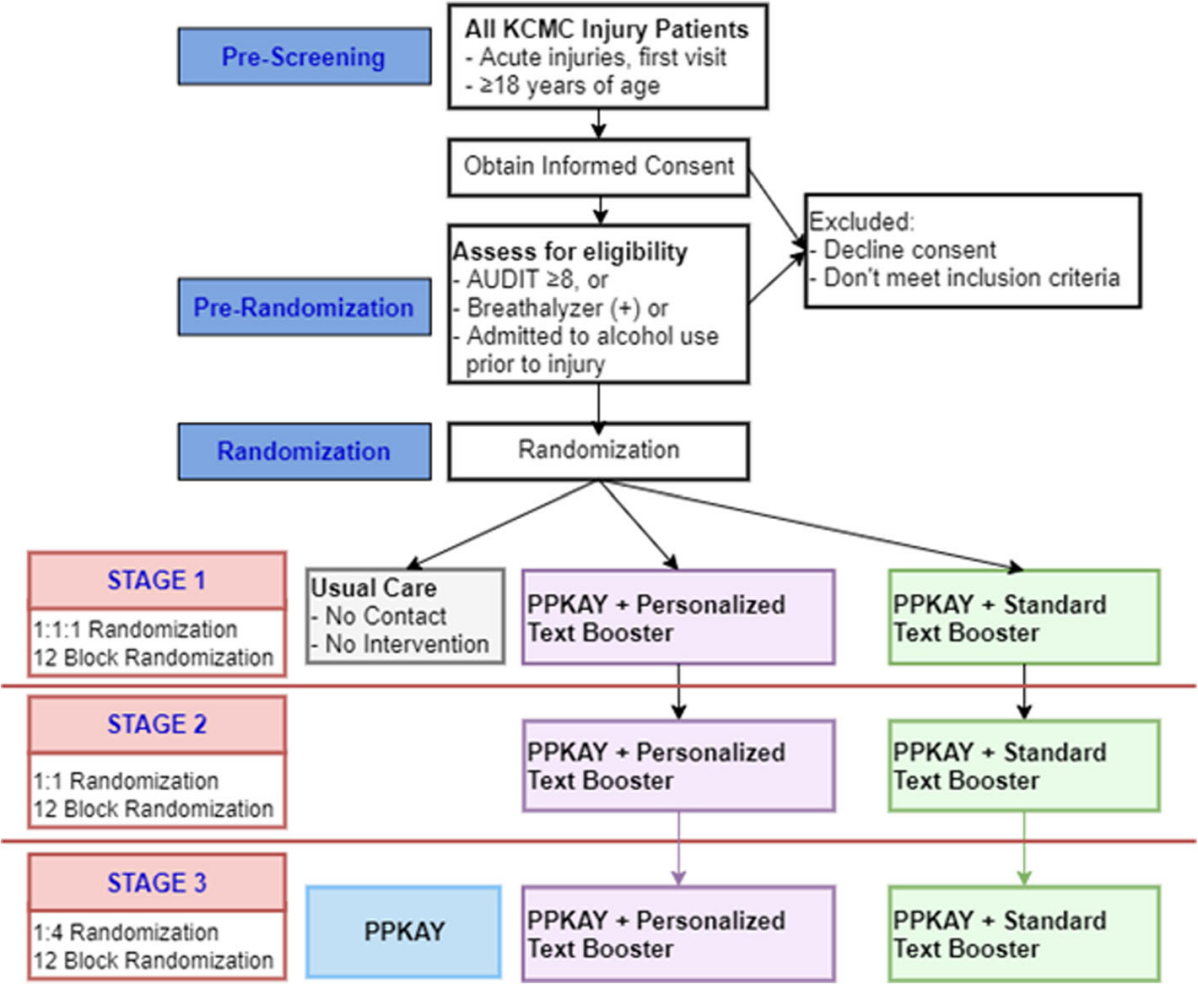
Diagram of the PRACT study design with trial conditions

This is a PRACT with three adaptive stages. Stage 1 includes a three-arm randomized controlled superiority trial comparing our culturally adapted BNI, “Punguza Pombe Kwa Afya Yako (PPKAY)” (Swahili: “Reduce Alcohol for Your Health”), with standard or personalized text booster to usual care (UC). After dropping the UC arm, Stage 2 will be a non-inferiority trial comparing the PPKAY with personalized and standard text booster. Stage 3, also a non-inferiority trial, compares the winner from Stage 2 against a new arm consisting of the PPKAY without a booster (Fig. 1). Enrollment for stages will be according to 12-block randomization at a 1:1:1 allocation for Stage 1, 1:1 allocation for Stage 2, and 1:4 allocation for Stage 3.

## Method

### Participants, interventions, and outcomes

#### Setting

The Kilimanjaro Christian Medical Centre (KCMC) in Moshi, Tanzania, is our selected low-resource setting. Tanzania is the most populated country in eastern Africa, with a population of approximately 52.5 million people [25]. Forty-five percent of the population identify as lifetime alcohol abstainers, while approximately 15% of the population admit to heavy periodic drinking [9, 26]. Studies have documented a very high proportion of patients with problematic drinking upwards of 15% and higher rates among high-risk groups (36% female bar workers and 47% male sex workers) [12]. Overall, the amount of alcohol consumed per capita in Tanzania has increased by more than 10% in the past 5 years [9, 26]. Moshi is a city in the Kilimanjaro region of northern Tanzania. The Moshi urban area has a population of over 180,000, while the larger Moshi area is home to approximately 466,000 people [25]. KCMC is the third-largest hospital in the country [27], the zonal referral center and a regional training center for all types of healthcare workers located in northeastern Tanzania. On average, the ED at KCMC sees 70–100 patients daily, of whom 30 are admitted. Annually, KCMC ED sees about 2,000 patients who suffer injuries, of whom approximately 30% have consumed alcohol prior to their injury. Alcohol use has been associated with a fivefold increase in the odds of injury and appears to have a dose-dependent effect on the odds of injury and violence at KCMC in Tanzania [28, 29].

#### Eligibility criteria—participants

Adult (≥18 years of age) patients will be eligible if they have suffered an acute injury (< 24 h) prior to presentation for care at KCMC Emergency Department. Included patients must disclose alcohol use prior to the injury, score ≥8 on the AUDIT, and/or test positive (> 0.0 g/dL) by alcohol breathalyzer. Participants need to be clinically sober at the time of enrollment and provide informed consent. Capacity to consent will be determined by a clinician based on medical history and physical examination. For patients who are severely ill or intoxicated upon arrival to the ED, reassessment will be conducted to check if they have regained the capacity to consent. Exclusion criteria include not speaking Swahili, not having access to a phone to receive SMS texts (including prisoners), or being an East Africa resident for less than 5 years.

#### Participant recruitment and follow-up

Patients will be recruited after being pre-screened for age, acute injury, exclusion criteria, and capacity to consent. Written informed consent will be acquired from all pre-screened patients interested in the trial. Research assistants will explain the information about the study arms and the content of the texts that will be received by the study participants and address any concerns about confidentiality during the consent process. Pre-randomization screening and determination of eligibility criteria will be conducted for patients who demonstrate the willingness to participate. During enrollment, at least two phone numbers will be identified and tested by participants for follow-up needs. Research team members blinded to allocation will contact participants during the follow-up period to arrange follow-up assessments. To ensure follow-up, if needed, the team members will travel to patients, provide financial support for their travel to the hospital, or conduct follow-ups over the phone. Previous engagement with this population has highlighted the need to include family phone numbers and other locator information with patient permission. Participants are not paid for participation but are reimbursed for their time at 5000 Tanzanian Shilling ($2.16) at each screening and follow-up period. The first enrollment was performed on 12 October 2020. The project is currently recruiting participants, and the expected end of data collection will be in February 2024.

#### Maximum sample size

##### Stage 1

An effective intervention for Stage 1 will be defined as having an effect size of at least 35% difference in the reduction in the primary outcome (number of binge drinking events per four weeks), between usual care and intervention arms combined. Assuming an average of four binge drinking days per month (SD = 3.4), a 35% difference translates into a difference of about 1.2 day reduction in the number of binge drinking days per month. Evidence from studies in high-income settings suggests that studies similar to Stage 1 have shown moderate to high treatment effects of 20–40% in the reduction of binge drinking days [11, 30–32]. A sample size of 205 patients per arm will have 80% power to be able to identify if the intervention arms are superior to usual care with a significance of 5%, and a conservative 80% retention rate.

##### Stages 2 and 3

A maximum of 365 participants per arm will be required for Stages 2 and 3. New enrollment numbers will be smaller, depending on the number of patients that are carried over from previous stages. This sample size will allow for testing the non-inferiority of PPKAY without text booster in comparison to the PPKAY + Personalized Text Booster, or the PPKAY + Standard Text Booster (whichever wins in Stage 2). This sample would be enough to identify non-inferiority with a plausible futility margin of 0.7 binge drinking day per month (half of the effect of the intervention against UC in Stage 1), assuming a standard deviation of the difference at 3.4, 80% power, 5% significance level, and 80% retention rate. Currently, there are no studies similar to Stage 2 or 3 for ED injury patients to model the effect size estimation. Since Stage 2 and 3 analyses will include patients enrolled from Stage 1, in contrast to a typical trial, there will be a smaller maximum enrollment per stage. For Stage 3, a 1:4 allocation is anticipated to increase the enrollment of patients in the PPKAY without SMS Booster arm, while the comparison arm (PPKAY + Personalized Text Booster or PPKAY + Standard Text Booster) carries over patients from Stages 1 and 2 (Table 1).

**Table 1:** Maximum enrollment numbers

		Usual care	PPKAY + Personalized Booster	PPKAY + Standard Booster	PPKAY	Enrollment per stage by sample size
Stage 1	Enrollment	205	205	205	N/A	615
	Total patients	205	205	205	N/A	
Stage 2	Enrollment	N/A	160	160	N/A	320
	Total patients	N/A	365	365	N/A	
Stage 3	Enrollment	N/A	91	N/A	365	456
	Total patients	N/A	456	N/A	365	
Maximum enrollment						1391

##### Adaptation plan

**Fig. 2:**
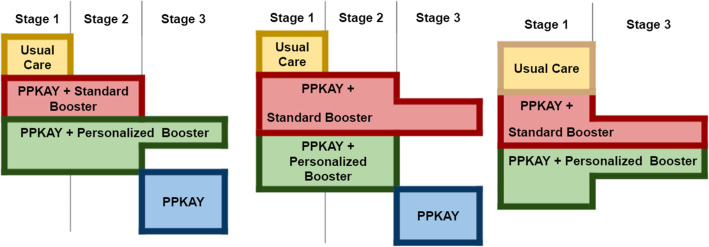
Scenarios of potential adaptations to the PRACT

Interim Analyses and Adaptations: In Stages 1 and 2, up to 3 interim analyses will be conducted after participants complete the 3 months of follow-up. At interim analyses, the PRACT will (1) continue the enrollment, (2) adapt to another stage according to predefined criteria, or (3) end the study for success or futility. Success in Stage 1 will be defined as the randomization to the intervention arm (PPKAY + Personalized Text Booster or PPKAY + Standard Text Booster) demonstrating superiority to UC in the difference in reduction of number of binge drinking days per month, with 80% power to identify the necessary effect at interim analysis. If a stopping rule is reached, we will progress to Stage 2 and stop enrollment in UC. Futility in Stage 1 will be defined if no statistical difference is detected after maximum enrollment (*N*=205 per arm) in Stage 1. If that happens, we will enter an exploratory stage where we evaluate the intervention among a restricted population and delineate mediators of the intervention’s impact, described further below (Fig. 2).

Assessing success in Stage 2 will be defined in non-inferiority evaluation of PPKAY + Standard Text Booster against PPKAY + Personalized Text Booster. Successful interim analyses for Stage 2 is defined as reaching a point where PPKAY + Standard Text Booster is shown to be non-inferior to PPKAY with personalized booster; then, the trial will discontinue the PPKAY + Personalized Text Booster arm and initiate enrollment to the PPKAY w/o Text Booster. However, if PPKAY + Standard Text Booster proves to be inferior to PPKAY + Personalized Text Booster, we will discontinue the PPKAY + Standard Text Booster arm.

In Stage 3, success will be defined in non-inferiority evaluation of PPKAY w/o Text Booster against the PPKAY arm that prevails in Stage 2. Successful interim analyses for Stage 3 are defined as reaching a point where PPKAY w/o Text Booster is shown to be non-inferior or inferior to PPKAY with Text Booster winner in Stage 2; then, the trial ends.

Possible adaptations through the three stages delineated above are depicted in Fig. 2. The third possible scenario would be if PPKAY + Standard Booster and PPKAY + Personalized Booster in Stage 1 is not superior to UC. In that situation, we will narrow the population eligible for enrollment to ensure we are identifying the optimal population for which this intervention might work. Given brief interventions in high-income settings are not as impactful, we plan to narrow our sample size to those with AUDIT score 8–18 only. We will perform an interim analysis to identify any signal of effectiveness among smaller sub-populations to inform this final adaptation.

##### Adaptive sample size

The sample size was calculated for a change in the primary outcome of reduction in the number of binge drinking days per month. Depending on the effect size found at each interim analysis, it is possible that significance could be reached earlier than expected. For Stage 1, recent literature supports effect sizes of 20–40% decrease. Currently, there is no intervention currently available for alcohol use reduction post injury in Tanzania. Through adaptive sample re-estimation and the most effective scenario, total minimum enrollment could be as low as 820 patients. Hypothesis testing was adjusted at interim analysis according to the O’Brien-Fleming alpha-spending function [33]. Thus, instead of spreading the alpha equally across interim analysis, the distribution is progressive leaving most of the significance levels later on the trial, when more patients are enrolled. If challenges arise with the adaptive nature of this trial, we will resort to standard sequential RCTs with maximum enrollment numbers. If standard RCT enrollment is used, an adequate sample size will be obtained to show the same effect size with a higher power.

### Assignment of interventions

#### Intervention

##### Brief intervention

Participants allocated to the BNI conditions will undergo a 15-minute discussion administered by a nurse. This intervention, based on the “Screening and Brief Intervention for Unhealthy Alcohol Use in the ED” [34] was linguistically and culturally adapted and piloted as the Punguza Pombe Kwa Afya Yako (PPKAY, “Reduce Alcohol for Your Health”) [35–41]. The nurse will initiate a four-step discussion: (1) raise the subject of alcohol, (2) provide feedback, (3) enhance motivation, and (4) negotiate and advise. An in-depth description of the intervention, its culturalization, and SMS booster message creation and feasibility testing is available elsewhere, but a brief description is included below.

Before PPKAY, screening will be conducted with questions about the quantity and frequency of alcohol use, validated Swahili AUDIT, and breathalyzer testing. Patients will then be approached by an intervention nurse and asked for permission to raise the subject of alcohol, as part of initiating the PPKAY. The patient’s current alcohol use will be discussed in the context of local and international safe-use guidelines. Next, the patient’s readiness to change using a validated 0–10 readiness ruler will be assessed [3–5, 41]. The patient’s specific reasons for changing behavior will be identified and compared with their readiness ruler response, highlighting self-efficacy and personal capacity for behavior change. Finally, the intervention nurse will negotiate a reduction in alcohol use, optimally advising a level which is lower than safe-use guidelines [6]. At the close of the PPKAY, the nurse will thank the patient for their openness in discussing alcohol, promoting self-efficacy and a good patient-provider relationship.

##### SMS booster messages

Participants allocated to the PPKAY + Booster conditions will be offered the same PPKAY process described above plus weekly text messages for 52 weeks [7, 8]. Participants allocated to the PPKAY + Personalized Booster condition will be offered the PPKAY and the booster process as described above with personalized rather than standard messages. During the PPKAY, the patient will identify their reasons for change, which will then be inserted into the motivational texts, which were previously outlined [9]. The texts will be entered in the text system on the day of the PPKAY to be assessed for accuracy in weekly research team quality control meetings. Nurses will be provided with feedback for the organization and the quality of the texts in these meetings. An example of the weekly personalized booster text message content, in English, is: “If you are going to drink alcohol, find a buddy to help you stay within safe limits and hold you accountable. You can reach your goal of having only two drinks with the help of a good friend,” in the case where the patient identified a goal of “having only two drinks.”

##### Control condition

The goal of this pragmatic trial is to understand the benefits of PPKAY and its combination with different kinds of boosters, compared with the current standard practice or UC, making UC the control condition. Follow-up in the control arm will be continued for the same time at the same periods as the intervention arms until study completion.

### Intervention fidelity

Our nurses were trained in motivational interviewing by the principal investigator (PI). Specifically, the nurses did mock interventions with other staff/PI and got direct personalized feedback based on the BNI assessment scale. To ensure intervention fidelity, a bilingual researcher with experience administering PPKAY will evaluate audiotaped interventions and give intermittent feedback to nurses on the intervention adherence to motivational interviewing guidelines and the BNI Assessment Scale adapted to the Tanzanian setting. If we find intervention protocol deviations, educational feedback will be provided to the nurses. During the feasibility trial, the nurses were assessed using this PPKAY Assessment Scale and have shown to have high-fidelity scores as measured by this scale.

### Outcomes

The primary outcome in this study is the percentage change in the number of binge drinking days from baseline to 3 months between study arms as assessed by timeline follow back (TLFB) methods. Binge drinking is defined as having five or more drinks on one occasion for men or four or more drinks for women.

As secondary outcomes, we will examine the number of alcohol-related consequences, quantity of alcohol consumed per week, frequency of drinking days, and mental health. Table 2 shows which measure will be used for each outcome. All outcomes will be measured at baseline and 3, 6, 9, 12, and 24 months after enrollment if we manage to adapt early.

**Table 2:** Trial outcome measures

Outcome	Measure	Description	Variables and source
Primary	Number of binge drinking days	Percentage change in the number of binge drinking days in the past two weeks	TLFB
Secondary	Change in alcohol-related consequences	Average change in alcohol-related consequences score	DrInC
	Amount of alcohol use per week	Average change in the amounts of alcohol consumed in the past two weeks	TLFB
	Frequency of drinking days	Percentage change in the number of drinking days	TLFB
	Alcohol dependence	Average change in the AUDIT scores	AUDIT
	Mental health	Average change in the PHQ-9scores	PHQ-9

### Data collection, management, and analysis

Participants across all conditions will be followed up for impact evaluation for up to two years. Outcome assessments for all trial arms will be evaluated at 3, 6, 9, 12, and possibly 24 months, based on the adaptation process. In case of finding a large effect size in Stage 1 and an early adaptation, the total follow-up period will be extended to two years. All the tools for measuring primary and secondary outcomes have been assessed previously for appropriate cultural and linguistic adaptation. Additionally, our preliminary work in the validation of scales and processes has confirmed the psychometric properties of these tools [10]. Our team has prior training in standardizing quantity and alcohol content with a validated standardized visual guide [11]. Three patient logs (screening, intervention, and follow-up) will be used for maintaining patient records. We will keep separate logs to capture patient data and maintain outcome assessor blinding. Alcohol-related outcome questions will be embedded in a broad health-related questionnaire to prevent reporting biases. All patient logs and follow-up data will be kept by research personnel on paper or entered into an online data repository, REDCap. Quality assurance processes will occur for paper collected data and computer (internet-based) data. At least 10% of the internet database will undergo data quality checks for completeness and consistency allowing for a recheck on the data collection and data entry process. Any quality issues will be addressed at each stage through further quality assurance education for the nurses. In terms of data monitoring We have established a Data Safety Monitoring Board (DSMB) made up of investigators from the United States and Tanzania who have expertise in mental health, alcohol treatment interventions in Tanzania, clinical trial management globally, and adaptive clinical trials. This DSMB is independent from the sponsor and is without any competing interests.

### Randomization

#### Sequence generation

The random number sequence generation at all stages will be conducted using computer software. Prior to study initiation, Stage 1 potential study identification numbers (SINs) will be randomized into three groups (UC, PPKAY + personalized Booster or PPKAY + Standard Booster) in 12-block randomization at 1:1:1 allocation. Potential Stage 2 SINs will be randomized into two groups (PPKAY + Personalized Booster or PPKAY + Standard Booster) in 12-block randomization at 1:4 allocation. Potential Stage 3 SINs will be randomized into two groups (the winner of Stage 2 or PPKAY without Booster) in 12-block randomization in 1:4 allocation, respectively. Randomization envelopes will be prepared for 110% of maximum enrollment numbers per stage. Block randomization will maintain balance among the study arms given the potential impact of time-dependent events impacting alcohol use (e.g., seasons, holidays, and payday).

#### Allocation concealment mechanism

Enrollment packets of the same size and thickness will be placed in opaque envelopes, sealed and locked in a drawer at the study site in consecutive order by study identification number. In each envelope there will be one paper delineating the random group assignment for each participant. To ensure reproducibility in any resourced context, paper randomization packets, and data collection sheets will be maintained.

#### Blinding

Intervention nurses will perform the intervention, while all other trial processes including screening, data collection, and follow-up assessments will occur by other research staff. Thereby, outcome assessors will be blinded to patients’ allocation. This will be managed through a clear delineation of roles. Investigators and research personnel will remain blinded whenever possible. Blinding will be removed only for the analysis team for the purpose of decision-making with regard to the adaptation to the next stage. The analysis lead cannot be fully blinded given the data management, quality control, and Stage 1 analysis for adaptation. However, the analysis lead will have minimal involvement in the trial at the study site in order to limit the influence on patients, interventionists, and outcome assessors.

### Statistical methods

Patient demographics will be compared across all arms of the study. Descriptive data will be reported as means, standard deviations, and frequencies. Outcome data will be reported as predicted means with confidence intervals for each follow-up time point (3, 6, 9, 12, and potentially 24 months). Missing data will be addressed with multiple imputation and inverse weighting with sequential sensitivity analysis to handle missing data. For the patients that are randomized to the intervention but do not adhere to the intervention, we will use an intention to treat approach.

Preliminary analyses will be conducted to evaluate baseline differences and differences in attrition across study arms. These comparisons will be made using Student’s *t*-test or Mann-Whitney *U* test according to the data distribution, and Pearson chi-squared test for categorical variables. We will test the primary hypothesis of the efficacy of the intervention using a longitudinal constrained approach (LCA), considering the outcome is the difference between baseline and follow-up. This approach allows for the prediction of follow-up scores after controlling for participants’ baseline characteristics and other potential confounders (potential predictors of attrition and outcome confounders). The models will be fit using a Poisson distribution, given the count nature of the primary outcome, as well as for secondary analysis using the frequency of drinking. Given our previous observational data, we expect that the data distribution will best fit a negative binomial method with a log link function, a recommended approach for over dispersed alcohol consumption data. For the AUDIT score, alcohol-related complications score, quantity of drinking, and mental health scale data, linear models will be used. For significant interaction terms, contrasts of marginal means will be conducted, and the Wald chi-square test statistic interpreted. The effect size will be reported as incidence risk ratios (for count data) and the difference in the predicted means (for numeric data).

#### Sub-study evaluation

Our estimated maximum sample per arm in Stage 1, Stages 2 and 3, would be sufficient to have 80% power to detect an *R*^2^ of 0.25 using generalized linear models to evaluate the moderation and/or mediation effect of our predefined set of biological, injury-related and alcohol use behavior-related indicators. Moderation analysis for categorical moderators (e.g., gender), analysis of covariance will be conducted to model post-intervention levels of dependent variable with the moderator and intervention arm condition as factors and baseline levels of the dependent variable as a covariate. For numeric moderators (e.g., age), generalized linear models will be used. Multivariate regression will test the main effects of intervention arm, moderator, and the interaction of intervention arm and moderator for each outcome group. For mediation analysis, a set of multiple mediation models will be conducted to determine whether these potential intermediary variables mediate the effect of PPKAY on outcomes and the extent to which individual variables will mediate the effect, conditional on the presence of other mediators in the model. The indirect effect of intervention on outcomes via each individual mediator will be the product of the path from intervention to mediator (a) and the path from mediator to outcome (b). The impact of all mediators included in the model will be expressed as the total indirect effect of intervention on outcome (a+b). Mediation models will be built using path analysis, with the addition of latent variables when applicable (e.g., AUDIT).

#### Dissemination

Important protocol modifications can be initiated by investigators or the DSMB and will flow to the other, thereafter will be amended in the appropriate regulatory bodies, trial registries, and study team. If there are important modifications to the trial, it will be reported in the trial results manuscripts. In accordance with institutional regulatory bodies (Duke University, Kilimanjaro Christian Medical Centre, and National Institute of Medical Research in Tanzania) the final trial dataset will be available to investigators with the use of a data use agreement and will be shared with the NIAAA data repository. All NIH-funded projects are compelled to share an anonymized version of the dataset collected and used as the basis for the analysis process. The data submission to the NIAAA repository follows a 6-month calendar, with data submissions following this agenda. The full study protocol has been published in ClinicalTrials.gov. De-identified participant-level data will be available on the NIAAA data repository, and any statistical analysis performed will be included in peer-reviewed manuscripts. Considering ancillary and post-trial care needs, it is very unlikely that any participant will suffer harm from this behavioral interventional trial. Study results will be presented at Kilimanjaro Christian Medical Centre, reported to the Tanzanian National Institute of Medical Research for dissemination, and published in national and international peer-reviewed publications.

See the corresponding SPIRIT Checklist (Supplementary File) for details.

## Discussion

This PRACT protocol aims to address the World Health Organization’s call to increase practice-based evidence in global mental health through the creation and validation of innovative interventions for low-resource settings [4, 9]. Specifically, by using a pragmatic randomized adaptive clinical trial, we posit that this trial will be both efficient and cost-effective in defining and demonstrating the effectiveness of PPKAY in our setting.

While effectiveness is yet to be determined, our PPKAY intervention has been thoughtfully linguistically and culturally translated and adapted from international guidelines to be most effective for the local context and patient population. Furthermore, this study is pragmatic through its implementation by nurses and with a specific focus on effectiveness in the Tanzanian clinical environment. The adaptive design of the trial will facilitate a streamlined patient enrollment and allow a change of focus from a less hierarchical medical model. Similarly, we have expanded our inclusion criteria from other international trials (i.e., admit to alcohol use prior to injury, AUDIT > 8 or breathalyzer + on arrival) to both fit the long prehospital times in our setting and our limited alternative treatment options for those with severe AUD (AUDIT > 18). These innovative differences can help us understand patient populations which might best be served with this intervention.

In light of the recent negative findings of brief interventions for ED patients during effectiveness trials in Europe, many have argued that the intervention fidelity is poor in those large effectiveness trials [10, 11, 42]. This is likely a concern across settings and anticipated in our setting and that of others with a very high clinical burden in EDs. To mitigate this concern, we have adopted a near-pragmatic approach where the intervention is administered by clinical ED nurses during low clinical volume periods and/or non-clinical ED nurses during high-volume periods. Similarly, we have adopted rigorous intervention fidelity and quality improvement processes in order to ensure fidelity during this trial as well as for our implementation plan.

We may identify further mediators or moderators to the PPKAYs effectiveness and expect additional improvements to maximize PPKAY’s positive effects in future settings. It is anticipated that this effectiveness data can support the incorporation of a brief negotiational intervention into the standard of care for injured patients in Tanzania and likely also in the broader region. Meanwhile, we hope that sharing this protocol will facilitate the implementation of other adaptive trials seeking to reduce the burden of alcohol use and related harm in low-resource settings.

## Trial Status

Trial Registration No.: NCT043535011

IRB Protocol No.: Pro000103724

Registration date: September 1, 2020

Actual study start date: October 12, 2020

Estimated study completion date: February 2024

Recruitment status: Recruiting

## Supplementary Information

**Additional file 1:.** SPIRIT 2013 Checklist: Recommended items to address in a clinical trial protocol and related documents*.

## Data Availability

The project database will be shared with the NIAAA, following anonymization routines to preserve the participant’s identity.

## Abbreviations

AUDIT: Alcohol Use Disorders Identification Test
BNI: Brief Negotiated Interview
DrInC: Drinker Inventory of Consequences
ED: Emergency department
KCMC: Kilimanjaro Christian Medical Centre
PI: Principal investigator
PPKAY: Punguza Pombe Kwa Afya Yako Swahili: “Reduce Alcohol for Your Health”
PRACT: Pragmatic, adaptive, randomized controlled trial
REDCap: Research Electronic Data Capture
SMS: Short-message-service
TLFB: Timeline follow back
UC: Usual care
