# Supplementary material for: PRACT: a pragmatic randomized adaptive clinical trial protocol to investigate a culturally adapted brief negotiational intervention for alcohol use in the emergency department in Tanzania
Source: Trials. 2022 Feb 5;23:120. doi: 10.1186/s13063-022-06060-y (PMC8818146; doi:10.1186/s13063-022-06060-y)
Supplement: Supplementary file 1 — Additional file 1:. SPIRIT 2013 Checklist: Recommended items to address in a clinical trial protocol and related documents*. [file 13063_2022_6060_MOESM1_ESM.doc]

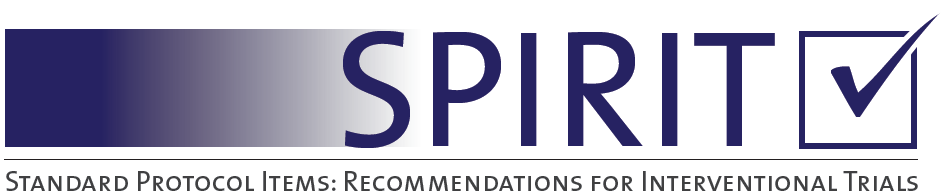


SPIRIT 2013 Checklist: Recommended items to address in a clinical trial protocol and related documents*

| Section/item | ItemNo | Description |
| --- | --- | --- |
| **Administrative information** | | |
| Title | 1 | Descriptive title identifying the study design, population, interventions, and, if applicable, trial acronym – **Section:** Title page – A PRACT: A Pragmatic Randomized Adaptive Clinical Trial Protocol to Investigate a Culturally Adapted Brief Negotiational Intervention for Alcohol Use in the Emergency Department in Tanzania |
| Trial registration | 2a | Trial identifier and registry name. If not yet registered, name of intended registry- **Section:**Trial registration - Page 4, Trial registration: NCT04535011, Registered September 1, 2020 |
|  | 2b | All 24 items from the World Health Organization Trial Registration Data Set   1. Primary Registry and Trial Identifying Number - **Section:**Trial registration - Page 4, Trial registration: NCT04535011, Registered September 1, 2020 2. Date of Registration in Primary Registry - **Section:**Trial registration - Page 4, Trial registration: NCT04535011, Registered September 1, 2020 3. Secondary Identifying Numbers - **Section:**Trial registration - Page 4, Trial registration: NCT04535011, Registered September 1, 2020 4. Source(s) of Monetary or Material Support – **Section:**Funding - Page 24, The current study is funded by the National Institute on Alcohol Abuse and Alcoholism (NIAAA) from the NIH, grant number: 1R01AA027512-01A1 5. Primary Sponsor - **Section:**Funding - Page 24, The current study is funded by the National Institute on Alcohol Abuse and Alcoholism (NIAAA) from the NIH, grant number: 1R01AA027512-01A1 6. Secondary Sponsor(s) – N/A 7. Contact for Public Queries - **Section:**Corresponding author – Page 2 – Corresponding author: Catherine A. Staton, TEL: (919)684-5537, FAX: 919-681-8521, Address: 2301 Erwin Road, DUMC Box 3096, email: catherine.lynch@duke.edu 8. Contact for Scientific Queries - **Section:**Corresponding author – Page 2 – Corresponding author: Catherine A. Staton, TEL: (919)684-5537, FAX: 919-681-8521, Address: 2301 Erwin Road, DUMC Box 3096, email: catherine.lynch@duke.edu 9. Public Title – **Section:**Title page – A PRACT: A Pragmatic Randomized Adaptive Clinical Trial Protocol to Investigate a Culturally Adapted Brief Negotiational Intervention for Alcohol Use in the Emergency Department in Tanzania 10. Scientific Title - **Section:**A PRACT: A Pragmatic Randomized Adaptive Clinical Trial Protocol to Investigate a Culturally Adapted Brief Negotiational Intervention for Alcohol Use in the Emergency Department in Tanzania 11. Countries of Recruitment – **Section:**Participants – Page 7 Adult (≥18 years of age) patients will be eligible if they have suffered an acute injury (<24 hours) prior to presentation for care at KCMC Emergency Department, Tanzania. 12. Health Condition(s) or Problem(s) Studied – **Section:**Introduction- Page 5 , text starting with: As such, we propose the PRACT to determine the effectiveness of this alcohol harm reduction intervention in three adaptive stages. 13. Intervention(s) – **Section:** Intervention – Page 12, Participants allocated to the BNI conditions will undergo a 15-minute discussion administered by a nurse. This intervention, based on the “Screening and Brief Intervention for Unhealthy Alcohol Use in the ED” Key Inclusion and Exclusion Criteria – Participants – page 7 paragraph starting at line 146: “Adult (≥18 years of age) patients will be…..” 14. Study Type - Method and design – Trial design, page 3, paragraph starting at line 116: “This is a PRACT with three adaptive stages” 15. Date of First Enrollment – Participant Recruitment and Follow-up, page 8 line 172. 16. Sample Size – Maximum Sample Size, page 9, Table 1, 199. 17. Recruitment Status – Participant Recruitment and Follow-up, page 8 line 172. 18. Primary Outcome(s) – Abstract, page 3 line 57: The primary outcome for the study…. 19. Key Secondary Outcomes – Abstract, page 3 line 58: Secondary outcomes include alcohol-related….. 20. Ethics Review – Declarations: Page 23 line 489. “Appropriate regulatory body approval has been obtained…” 21. Completion date – Participant Recruitment and Follow-up, page 8 line 173. 22. Summary Results – N/A 23. IPD sharing statement – Dissemination – Page 20 paragrah starting at line 419: “Important protocol modifications can be initiated by investigators….” |
| Protocol version | 3 | Date and version identifier- **Section:** Protocol version, Page 2, Protocol version: version 1, date of first submission September 1, 2020. |
| Funding | 4 | Sources and types of financial, material, and other support - **Section:** Funding - Page 24, The current study is funded by the National Institute on Alcohol Abuse and Alcoholism (NIAAA) from the NIH, grant number: 1R01AA027512-01A1. The PI of the project funded is Catherine Staton. |
| Roles and responsibilities | 5a | Names, affiliations, and roles of protocol contributors- **Section:**Authors , Page 1, line 7. |
|  | 5b | Name and contact information for the trial sponsor- Page 2, line 33. Laura Elizabeth Kwako, laura.kwako@nih.gov |
|  | 5c | Role of study sponsor and funders, if any, in study design; collection, management, analysis, and interpretation of data; writing of the report; and the decision to submit the report for publication, including whether they will have ultimate authority over any of these activities- **Section:**Funding – Page 24, line 512: The funding body has no responsibility in terms of the design, data collection, analysis, interpretation of data, and in writing of the present manuscript. |
|  | 5d | Composition, roles, and responsibilities of the coordinating centre, steering committee, endpoint adjudication committee, data management team, and other individuals or groups overseeing the trial, if applicable (see Item 21a for data monitoring committee) - **Section:**Data Collection – Page 17, We have established a Data Safety Monitoring Board (DSMB) made up of investigators from the United States and Tanzania who have expertise in mental health, alcohol treatment interventions in Tanzania, clinical trial management globally and adaptive clinical trials. |
| Introduction |  |  |
| Background and rationale | 6a | Description of research question and justification for undertaking the trial, including summary of relevant studies (published and unpublished) examining benefits and harms for each intervention – **Section:** Background and rationale – Page 4 up to Page 5. |
|  | 6b | Explanation for choice of comparators- **Section:** Background and rationale – Page 6 – paragraph starting with: This is a PRACT with three adaptive stages. Stage 1 includes a three-arm randomized controlled superiority trial comparing our culturally. |
| Objectives | 7 | Specific objectives or hypotheses- **Section:** Background and rationale – Page 5 – text starting with: As such, we propose the PRACT to determine |
| Trial design | 8 | Description of trial design including type of trial (eg, parallel group, crossover, factorial, single group), allocation ratio, and framework (eg, superiority, equivalence, noninferiority, exploratory) - **Section:** Background and rationale – Page 6, text starting with: This is a PRACT with three adaptive stages |
| Methods: Participants, interventions, and outcomes | | |
| Study setting | 9 | Description of study settings (eg, community clinic, academic hospital) and list of countries where data will be collected. Reference to where list of study sites can be obtained- **Section:**Participants, interventions, and outcomes – Setting, Page 6, text starting with: The Kilimanjaro Christian Medical Centre (KCMC) in Moshi, |
| Eligibility criteria | 10 | Inclusion and exclusion criteria for participants. If applicable, eligibility criteria for study centres and individuals who will perform the interventions (eg, surgeons, psychotherapists) - **Section:**Participants, interventions, and outcomes – Eligibility criteria - Page 7, text starting with: Adult (≥18 years of age) patients will be eligible if they have |
| Interventions | 11a | Interventions for each group with sufficient detail to allow replication, including how and when they will be administered- **Section:**Participants, interventions, and outcomes – Page 12: text starting with: Intervention  Brief Intervention  Participants allocated to the BNI conditions will undergo a 15-minute discussion administered by a nurse. This intervention |
|  | 11b | Criteria for discontinuing or modifying allocated interventions for a given trial participant (eg, drug dose change in response to harms, participant request, or improving/worsening disease) – **Section:**Participants, interventions, and outcomes – Adaptation plan, Page 11, text starting with: Assessing success in Stage 2 will be defined in non-inferiority evaluation of PPKAY + Standard Text Booster |
|  | 11c | Strategies to improve adherence to intervention protocols, and any procedures for monitoring adherence (eg, drug tablet return, laboratory tests) - **Section:**Participants, interventions, and outcomes – Intervention Fidelity, Page 14, text starting with: Our nurses were trained in motivational interviewing by the principal investigator (PI). Specifically, the nurses |
|  | 11d | Relevant concomitant care and interventions that are permitted or prohibited during the trial- **Section:** Participants, interventions, and outcomes – Page 14, line 293. |
| Outcomes | 12 | Primary, secondary, and other outcomes, including the specific measurement variable (eg, systolic blood pressure), analysis metric (eg, change from baseline, final value, time to event), method of aggregation (eg, median, proportion), and time point for each outcome. Explanation of the clinical relevance of chosen efficacy and harm outcomes is strongly recommended- **Section:**Participants, interventions, and outcomes – Page 15, text starting with: The primary outcome in this study is the percentage change in the number of binge drinking days from baseline to 3 months between study arms as assessed by timeline follow back (TLFB) methods. |
| Participant timeline | 13 | Time schedule of enrolment, interventions (including any run-ins and washouts), assessments, and visits for participants. A schematic diagram is highly recommended (see Figure) - **Section:**Participants, interventions, and outcomes – Participant Recruitment and Follow-up , Page 8, text starting with: The first enrollment was performed |
| Sample size | 14 | Estimated number of participants needed to achieve study objectives and how it was determined, including clinical and statistical assumptions supporting any sample size calculations- **Section:**Participants, interventions, and outcomes – Page 8, text starting with: Maximum Sample Size  Stage 1: An effective intervention for Stage 1 |
| Recruitment | 15 | Strategies for achieving adequate participant enrolment to reach target sample size- **Section:**Participants, interventions, and outcomes – Participant Recruitment and Follow-up – Page 7, text starting with Participant Recruitment and Follow-up  Patients will be recruited after being pre-screened for age, acute injury, exclusion criteria, and capacity to consent |
| **Methods:** **Assignment of interventions (for controlled trials)** | | |
| Allocation: |  |  |
| Sequence generation | 16a | Method of generating the allocation sequence (eg, computer-generated random numbers), and list of any factors for stratification. To reduce predictability of a random sequence, details of any planned restriction (eg, blocking) should be provided in a separate document that is unavailable to those who enrol participants or assign interventions- **Section:**Methods: Assignment of interventions, Randomization  Sequence generation, Page 17, starting text with: The random number sequence generation at all stages will be conducted using computer software. Prior to study initiation, Stage 1 potential study identification numbers (SINs) |
| Allocation concealment mechanism | 16b | Mechanism of implementing the allocation sequence (eg, central telephone; sequentially numbered, opaque, sealed envelopes), describing any steps to conceal the sequence until interventions are assigned- **Section:**Methods: Assignment of interventions, Allocation concealment mechanism, Page 17, text starting with: Enrollment packets of the same size and thickness will be placed in opaque envelopes, sealed and locked in a drawer |
| Implementation | 16c | Who will generate the allocation sequence, who will enrol participants, and who will assign participants to interventions- **Section:** Methods: Assignment of interventions Blinding, Page 18, text starting with: Blinding  Intervention nurses will perform the intervention, while all other trial processes including screening, |
| Blinding (masking) | 17a | Who will be blinded after assignment to interventions (eg, trial participants, care providers, outcome assessors, data analysts), and how- **Section:**Methods: Assignment of interventions Blinding, Page 18, text starting with: Blinding  Intervention nurses will perform the intervention, while all other trial processes including screening, |
|  | 17b | If blinded, circumstances under which unblinding is permissible, and procedure for revealing a participant’s allocated intervention during the trial- **Section:** Methods: Assignment of interventions Blinding, Page 18, text starting with: Blinding  Intervention nurses will perform the intervention, while all other trial processes including screening, |
| **Methods:** **Data collection, management, and analysis** | | |
| Data collection methods | 18a | Plans for assessment and collection of outcome, baseline, and other trial data, including any related processes to promote data quality (eg, duplicate measurements, training of assessors) and a description of study instruments (eg, questionnaires, laboratory tests) along with their reliability and validity, if known. Reference to where data collection forms can be found, if not in the protocol - **Section:** **Methods:** Data collection, management, and analysis, page 16, text starting with: Participants across all conditions will be followed up for impact evaluation for up to two years. Outcome assessments for all trial arms will be evaluated at 3, 6, 9, 12 and possibly 24 months, based |
|  | 18b | Plans to promote participant retention and complete follow-up, including list of any outcome data to be collected for participants who discontinue or deviate from intervention protocols- **Section:**Participants, interventions, and outcomes, Participant Recruitment and Follow-up, page 8, text starting with: During enrollment, at least two phone numbers will be identified and tested by participants for follow-up needs. |
| Data management | 19 | Plans for data entry, coding, security, and storage, including any related processes to promote data quality (eg, double data entry; range checks for data values). Reference to where details of data management procedures can be found, if not in the protocol - Page 16, text starting with: **Section:**Data collection, management, and analysis , Quality assurance processes will occur for paper collected data and computer (internet-based) data. At least 10% of the internet database |
| Statistical methods | 20a | Statistical methods for analysing primary and secondary outcomes. Reference to where other details of the statistical analysis plan can be found, if not in the protocol- **Section:**Statistical Methods, page 18, text starting with: Patient demographics will be compared across all arms of the study. Descriptive data will be reported as means |
|  | 20b | Methods for any additional analyses (eg, subgroup and adjusted analyses) - **Section:**Sub-study evaluation, Page 19, text starting with: Our estimated maximum sample per arm in Stage 1, Stages 2 and 3, would be sufficient to have 80% power to detect an R2 of 0.25 |
|  | 20c | Definition of analysis population relating to protocol non-adherence (eg, as randomised analysis), and any statistical methods to handle missing data (eg, multiple imputation) – Section Statistical methods, page 18, line 382. |
| **Methods: Monitoring** | | |
| Data monitoring | 21a | Composition of data monitoring committee (DMC); summary of its role and reporting structure; statement of whether it is independent from the sponsor and competing interests; and reference to where further details about its charter can be found, if not in the protocol. Alternatively, an explanation of why a DMC is not needed - **Section:**Data collection, management, and analysis, Page 16, text starting with: In terms of data monitoring We have established a |
|  | 21b | Description of any interim analyses and stopping guidelines, including who will have access to these interim results and make the final decision to terminate the trial- **Section:**Adaptation Plan, page 10, text starting with: Interim Analyses and Adaptations: In Stages |
| Harms | 22 | Plans for collecting, assessing, reporting, and managing solicited and spontaneously reported adverse events and other unintended effects of trial interventions or trial conduct- **Section:**Data collection, management, and analysis, Page 16, text starting with: In terms of data monitoring We have established a |
| Auditing | 23 | Frequency and procedures for auditing trial conduct, if any, and whether the process will be independent from investigators and the sponsor - **Section:** Data collection, management, and analysis, Page 16, text starting with: At least 10% of the internet |
| Ethics and dissemination | | |
| Research ethics approval | 24 | Plans for seeking research ethics committee/institutional review board (REC/IRB) approval- **Section:**Ethics approval and consent to participate, Page 23, text starting with: Appropriate regulatory body approval has been obtained from the Kilimanjaro Christian Medical University College Ethics Committee (Certificate #2457) |
| Protocol amendments | 25 | Plans for communicating important protocol modifications (eg, changes to eligibility criteria, outcomes, analyses) to relevant parties (eg, investigators, REC/IRBs, trial participants, trial registries, journals, regulators) – **Section:** Dissemination, Page 20, Text starting with: Important protocol modifications can be initiated by investigators or the DSMB and will flow to the other, thereafter will be amended |
| Consent or assent | 26a | Who will obtain informed consent or assent from potential trial participants or authorised surrogates, and how (see Item 32) - **Section:**Participant Recruitment and Follow-up, Page 8, text starting with: Patients will be recruited after being pre-screened for age, acute injury, exclusion criteria, and capacity to consent. |
|  | 26b | Additional consent provisions for collection and use of participant data and biological specimens in ancillary studies, if applicable – On the consent form, participants will be asked if they agree to use of their data should they choose to withdraw from the trial. Participants will also be asked for permission for the research team to share relevant data with people from the Universities taking part in the research or from regulatory authorities, where relevant. This trial does not involve collecting biological specimens for storage |
| Confidentiality | 27 | How personal information about potential and enrolled participants will be collected, shared, and maintained in order to protect confidentiality before, during, and after the trial- **Section:**Data collection, management, and analysis, Page 16, text starting with: All patient logs and follow-up data will be kept by research personnel on paper or entered into an online data repository, REDCap. |
| Declaration of interests | 28 | Financial and other competing interests for principal investigators for the overall trial and each study site- **Section:**Competing interests, Page 24. |
| Access to data | 29 | Statement of who will have access to the final trial dataset, and disclosure of contractual agreements that limit such access for investigators- **Section:**Data collection, management, and analysis, Page 16, text starting with: In terms of data monitoring We have established a |
| Ancillary and post-trial care | 30 | Provisions, if any, for ancillary and post-trial care, and for compensation to those who suffer harm from trial participation- **Section:**Dissemination , Page 21, text starting with: Considering ancillary and post-trial care needs, it is very unlikely that any participant will suffer harm from this behavioral interventional trial. |
| Dissemination policy | 31a | Plans for investigators and sponsor to communicate trial results to participants, healthcare professionals, the public, and other relevant groups (eg, via publication, reporting in results databases, or other data sharing arrangements), including any publication restrictions – **Section:**Dissemination, Page 20, text starting with: the final trial dataset will be available to investigators with the use of a data use agreement and will be shared with the NIAAA data repository. |
|  | 31b | Authorship eligibility guidelines and any intended use of professional writers- Page 24-26. |
|  | 31c | Plans, if any, for granting public access to the full protocol, participant-level dataset, and statistical code - **Section:**Dissemination, Page 20, text starting with: the final trial dataset will be available to investigators with the use of a data use agreement and will be shared with the NIAAA data repository. |
| Appendices |  |  |
| Informed consent materials | 32 | Model consent form and other related documentation given to participants and authorised surrogates – **Section:** Attached as an supplementary document to the submission. |
| Biological specimens | 33 | Plans for collection, laboratory evaluation, and storage of biological specimens for genetic or molecular analysis in the current trial and for future use in ancillary studies, if applicable - There will be no biological specimens collected |

*It is strongly recommended that this checklist be read in conjunction with the SPIRIT 2013 Explanation & Elaboration for important clarification on the items. Amendments to the protocol should be tracked and dated. The SPIRIT checklist is copyrighted by the SPIRIT Group under the Creative Commons “[Attribution-NonCommercial-NoDerivs 3.0 Unported](http://www.creativecommons.org/licenses/by-nc-nd/3.0/)” license.
